# An Innovative Absorption Propagation System Hollow Block Made of Concrete Modified with Styrene–Butadiene Rubber and Polyethylene Terephthalate Flakes to Reduce the Propagation of Mechanical Vibrations in Walls

**DOI:** 10.3390/ma16145028

**Published:** 2023-07-16

**Authors:** Maciej Major, Izabela Adamczyk, Jarosław Kalinowski

**Affiliations:** Faculty of Civil Engineering, Czestochowa University of Technology, Dabrowskiego 69, 42-201 Częstochowa, Poland; izabela.adamczyk@pcz.pl (I.A.); jaroslaw.kalinowski@pcz.pl (J.K.)

**Keywords:** innovative concrete wall hollow block, reduction in mechanical vibrations

## Abstract

This paper discusses an innovative APS hollow block wall with a frame made of concrete modified with recycled materials. The technical data of the hollow block, the percentages of the recycled materials, including SBR rubber granules and PET flakes in the modified concrete, and the composition of the concrete modified with this mixture of recycled additives, are presented. To demonstrate the effectiveness of the solution in reducing mechanical vibrations, the effect of the interaction of different frequencies of the mechanical wave on reducing these vibrations was evaluated for APS blocks and Alpha comparison blocks. The test was carried out on a developed test stand dedicated to dynamic measurements for sixteen frequencies in the range from 8 to 5000 Hz, forcing a sinusoidal course of vibrations. The results are presented graphically and show that the new type of APS hollow block wall was much more effective in reducing mechanical vibrations. This efficiency was in the range from 10 to 51% for 12 out of the tested 16 frequencies. For the frequencies of 8, 16, 128, and 2000 Hz, the values were obtained with a difference of 3.58% in favor of the APS hollow block. In addition, the study of the damping effectiveness of the APS hollow blocks, in relation to the vibrations generated by an M-400 impact mill, showed that the APS block wall had a higher damping efficiency of 16.87% compared to the Alpha hollow block for the signal reading on the floor next to the mill, and 18.68% for the signal reading on the mill body. The modified concrete used in the production of the APS hollow blocks enabled the effective use of two recycled materials, SBR rubber and polyethylene terephthalate, in the form of PET flakes.

## 1. Introduction

Commonly used concrete wall hollow blocks are used for erecting the exterior and interior structural walls of buildings, especially underground floors. Their role in the construction of the wall, like that of clay blocks, consists of the transfer of vertical loads to the foundation [1,2]. This is provided by the concrete frame of the hollow block, which usually has alternately shaped through-holes. There are studies in the literature that have examined concrete with the addition of SBR (styrene–butadiene rubber) granules obtained from the processing of used car tires [3,4,5,6], polyethylene terephthalate in the form of PET (Poli(EtylenoTereftalanu)) flakes, or granules from used plastic bottles [7,8,9,10,11], but few publications have studied concrete modified with these two additives [12,13,14]. Contemporary concrete mix designs take into account waste management [8,12,15,16,17] and the use of “substitutes” that could replace some of the components needed to produce a cement matrix composite material. This is an important aspect of sustainable building [18]. Environmental protection is an important issue that should be taken into account in scientific studies. Examples of such activity may be seen in publications [19,20,21,22].

Aggregate in concrete mixes is a natural resource and requires massive mining when producing concrete on an industrial scale. Therefore, it becomes expedient to use other aggregate substitutes, and such research is also being conducted [17,23,24,25,26]. It is possible to reduce the cost of concrete production [27,28] and obtain a material with a modified matrix with specially designed properties [9,13,29,30,31,32]. This allows for expanding the applicability of the concrete matrix materials in various areas of industrial activity [23,31,33]. It is also worth mentioning the use of recycled materials in the production of cement [34].

Externally induced vibrations can be transmitted through a building structure from walls to ceilings. Vibration-reducing insulation placed in the through-holes of hollow blocks can improve their damping properties. In this way, the thermal insulation properties of a wall can also be improved [6,35]. Safeguards for hollow block wall construction have been discussed in studies [36,37,38,39,40,41,42,43,44,45], while the problem of improving the sound insulation of ceilings was described in [46,47]. The reduction in mechanical vibrations is a problem of importance for the safety of a structure and for the comfort of rooms intended for human habitation [48,49]. The authors showed that the use of damping materials in ceramic ceiling blocks and insulating materials in masonry walls significantly improves the comfort of these rooms.

The problem of moisture in the walls of floors located below ground level has been analyzed by many authors, including those of the studies in [50,51,52]. Soil, which is an elastic medium with a high density, allows for the propagation of mechanical vibrations induced by the operation of machinery or equipment and wheeled transport vehicles [53]. These problems were described in detail in [54,55,56,57,58]. Based on these studies, this problem is significant and still effectively unresolved. The phenomena associated with the propagation of a disturbance in the form of mechanical vibrations are wave-like in nature, since they lead to the vibration of the particles of the medium through which they propagate. The disturbance wave caused by a mechanical vibration is transmitted through the ground medium to the wall structure, then to the structure of the ceilings of individual stories. Similarly, the forces created by a change in sound pressure have a similar effect. Consequently, an acoustic wave with sufficiently high energy can induce vibrations in the particles of a building’s outer wall and move into the interior of the building, causing or amplifying noise [51,52,59]. Maintaining proper comfort in the rooms of buildings intended for human habitation necessitates the use of plate vibration isolation materials [60] for walls located below ground level, and vibroacoustic materials [61] for walls located above ground level. In publications on the impact of wheeled transport on the comfort of the use of enclosed buildings [55,56], authors have shown that it is sometimes necessary to use ground screens to reduce the mechanical vibrations caused by road and rail vehicles. This is especially true in urbanized areas of cities, where wheeled transport is steadily increasing year after year [62]. There are works referring to the area of the research issues undertaken in the paper [63,64,65,66].

The paper discusses a new type of concrete hollow wall block [67], which allows for an effective reduction in the mechanical vibrations inside the block. The efficiency of reducing the mechanical vibrations in this solution is ensured by a properly shaped support frame in the block made of concrete modified with recycled materials, as well as curvilinear through-holes and locks. This translates into a smaller wall thickness compared to the previously commonly used technological and material solutions that require additional slab insulation, i.e., vibration isolation.

## 2. APS Innovative Concrete Wall Hollow Block

**Figure 1:**
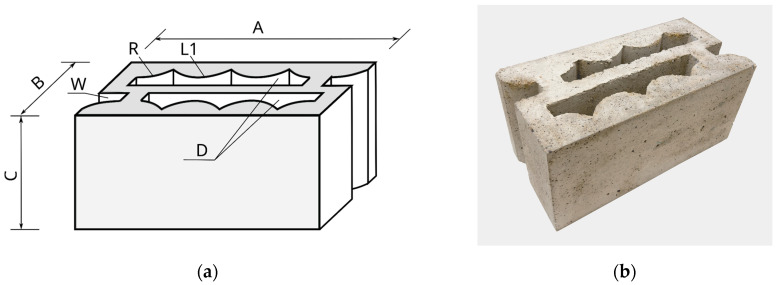
A new type of APS concrete wall block with non-linear through-holes and butt locks. (**a**) the shape: A—length, B—width, C—height, D—through-holes, R—radius of curvature of the through hole, L1—arc, and W—groove; (**b**) an example of the APS hollow block.

An APS (absorption propagation system) block is a cuboid with dimensions of A × B × C, which are 490 × 240 × 240 mm, respectively, with curvilinear through-holes and locks, according to Figure 1. The shape and construction of the new type of concrete wall hollow block are protected by patent PL 235427 B1 [67]. The actual dimensions and dimensional deviations were determined based on the standards in [68,69]. Permissible dimensional deviations are within category D1 of the standard [68] and amount to +3/−5 mm for length, width, and height. Standards [68,69] also specify the shape conditions to be met by a concrete masonry element, i.e., they allow such an element to have indentations, a joint system, roundness, or sharp edges. All these conditions are met by a new type of concrete hollow block—the APS.

**Figure 2:**
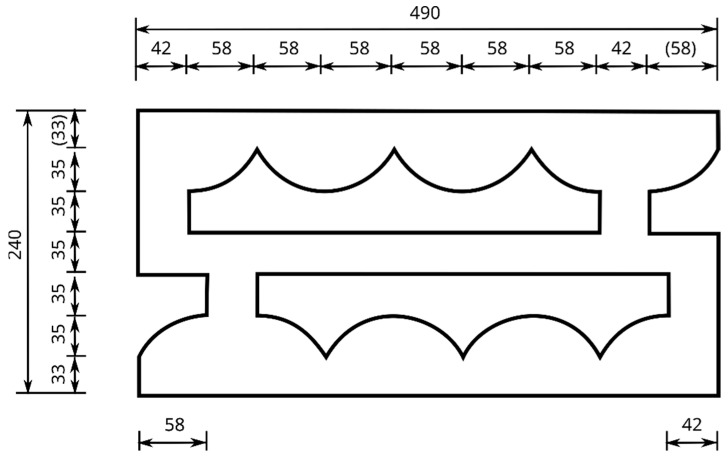
Horizontal cross-section of the developed hollow concrete wall block [mm].

Located inside the block, the through-holes D are designed in such a way that it is possible to reduce mechanical vibrations by dissipating the propagating mechanical wave directly in the block as a result of its multiple reflections. The through-holes D are located inside the block throughout its height C and described by an arc L1 with a radius R with central symmetry. On the side of the hollow block with a width B are grooves W. The APS block is designed to allow for a combination of the side-by-side placement of the blocks, rotated by 180 degrees alternately. The detailed dimensions in the horizontal section of the developed hollow concrete wall block are shown in Figure 2, while an example of the APS hollow block is shown in Figure 1b.

**Figure 3:**
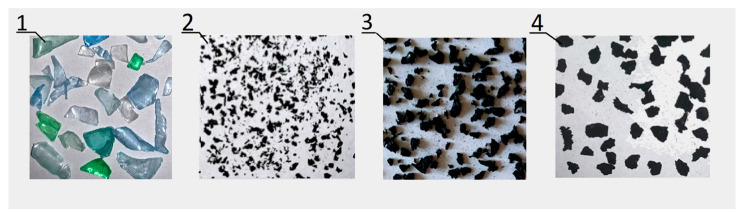
Recycling materials: 1—PET flakes, 2—SBR rubber granulate 0 ÷ 1 mm, 3—SBR rubber granulate 0.8 ÷ 2 mm, and 4—SBR rubber granulate 2 ÷ 4 mm.

Below are the technical data of the new type of APS block—Table 1, the percentage shares of the SBR rubber granulates and PET flakes used in the modified concrete of the APS block—Table 2, and the composition of the concrete modified with a mixture of recycling additives: SBR rubber granulate and PET flakes for a volume of 1 m^3^ of the concrete mix for the APS block—Table 3. Photos showing the materials used in the tests are presented in Figure 3.

**Figure 4:**
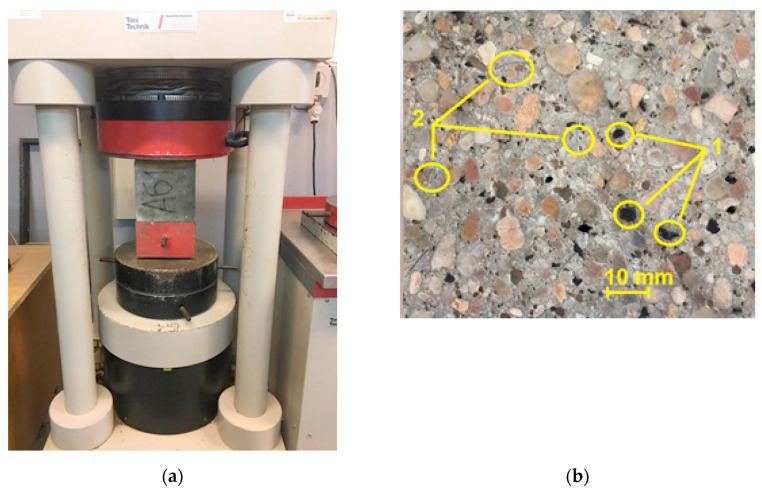
(**a**) Testing the compressive strength of a concrete sample using a Toni Technik ZWICK 2030 testing machine (manufactured: ZwickRoell, Ulm, Germany); (**b**) section of a sample of modified concrete of APS blocks after the test: 1—SBR rubber granulate, and 2—PET flakes.

The compressive strength test for the modified concrete of the APS hollow block was carried out on cubic samples perpendicular to the forming direction and centrally in relation to the centered pressure plates of the compression press, using a Toni Technik ZWICK type 2030 testing machine (Figure 4a) that meets the standard requirements PN-EN 12390-4 [71]. A constant sample loading rate of 1.0 MPa/s was used. The distribution of the additive mix in the modified concrete of the APS hollow blocks, obtained from the sample after the test, is shown in Figure 4b.

The average compressive strengths obtained in the test and the corresponding strength classes are presented in Table 4.

The compressive strength of the APS hollow block is given in Table 1.

The target concrete mix used to make the APS block was selected in the design process (experimental method), in which control concretes were designed at the beginning. After their modification with a mixture of recycling additives, taking into account various amounts of SBR granulate fraction, the target composition was selected, according to Table 3, the most effective in terms of the compressive strength.

**Figure 5:**
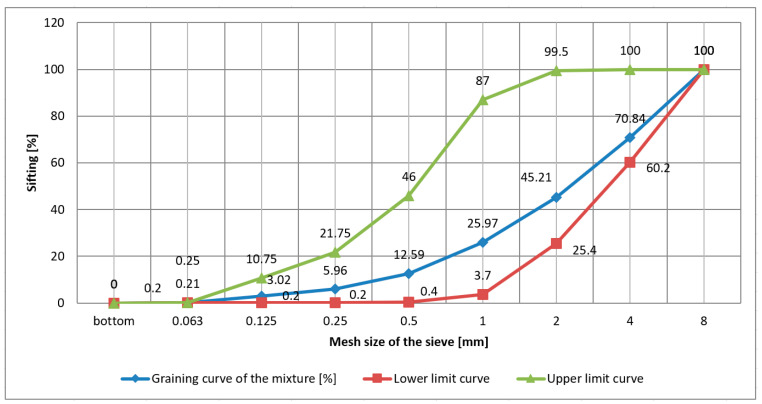
Graining curve of the composed aggregate mix for concrete mixes.

Based on the calculated percentages of the individual fractions of the composed aggregate mixture, a grain size curve was plotted between the limiting curves (Figure 5).

## 3. Recycling SBR and PET Materials

The use of recycling materials in the APS block included SBR rubber granulate and PET flakes in the amounts indicated in Table 2. The rubber granules were obtained from the processing of used car tires, while the PET flakes were obtained from used plastic bottles. The physical and chemical properties of the SBR rubber granules used in the tests are listed in Table 5. The basic physical and chemical properties of the polyethylene terephthalate in the form of PET flakes are presented in Table 6.

## 4. Comparative Alpha Hollow Block

**Figure 6:**
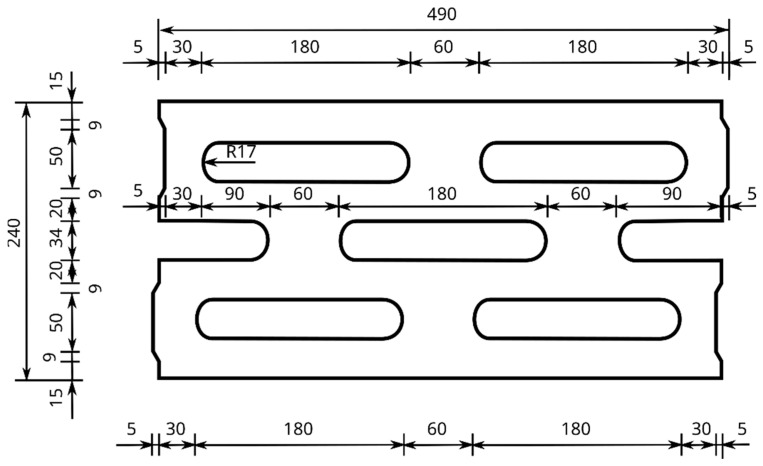
Horizontal cross-section of the comparative Alpha block.

**Figure 7:**
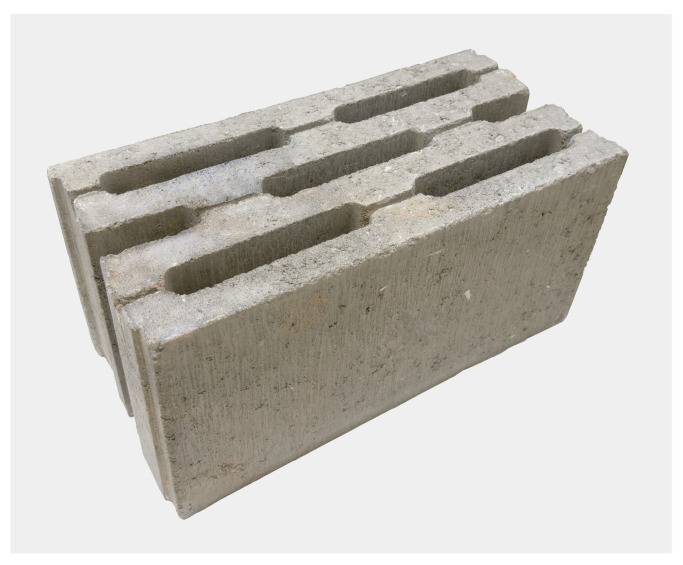
An example of the Alpha hollow block.

An Alpha 25 concrete hollow block was used as a comparison for the mechanical vibration reduction tests. It was purchased from the C.J. Blok Sp. z o. o. from Głogów Małopolski, Poland. The comparative hollow block accepted for testing had external dimensions of length × width × height, respectively, equal to 490 × 240 × 240 mm. These dimensions were identical to the dimensions of the new type of APS hollow concrete block. The area of the through-holes in the Alpha block was 1176 cm^2^ and was also comparable to the area of the holes in the new APS block, which was 1124 cm^2^. The basic dimensions of the Alpha block are shown in Figure 6 [74], while an example of the Alpha hollow block is shown in Figure 7.

Alpha concrete hollow blocks are intended for the construction of foundation walls in land and water construction. They meet the conditions of standard EN 771-3:2011 + A1:2015 [68]. The basic technical data of the Alpha block are presented in Table 7 [74].

## 5. Research of the Reduction in Mechanical Vibrations

Mechanical waves are generated by the interaction of at least two bodies and also arise during the vibration of a medium [75]. The impact caused by the movement of machinery or equipment is also of a wave nature and is transmitted through the ground, for example, to the foundations of buildings [76,77,78]. Consequently, mechanical vibrations are generated, causing the movement of the medium in which they were generated [79,80]. This results in the transmission of mechanical energy and its propagation in the building structure. This has a negative impact not only on the building, but also on the people inside the building. To reduce these mechanical vibrations propagating through the soil medium to the foundation walls, a new type of concrete wall hollow block, APS, was designed [67]. It is a hollow block with a modified recycled concrete frame and a patented solution for its internal structure, with curvilinear through-holes and locks.

**Figure 8:**
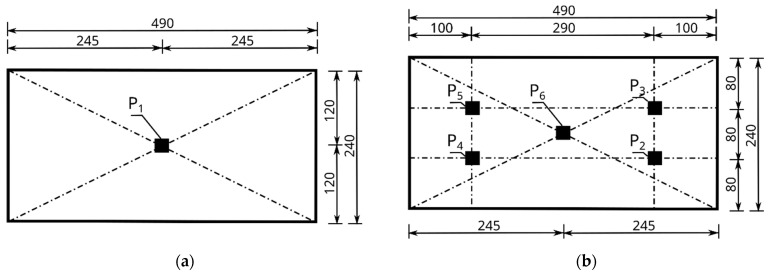
Scheme of arrangement of accelerometers on the walls of the tested hollow concrete wall blocks: (**a**) front wall with the location of the P_1_ accelerometer and the K2007E01 electromagnetic force exciter, (**b**) rear wall with the location of accelerometers P_2_ to P_6_. Dimensions are in mm.

The measurement of vibrations allows for the observation of complex, non-sinusoidal waveforms, which, after a frequency analysis, can be presented in the form of an amplitude spectrum. For a quantitative description of these vibrations, peak-to-peak values are taken, from which it is possible to determine the maximum value *q_imax_* of the difference in the positive deviation *qi_(+)_* and negative deviation *qi_(-)_* of the signal at the measurement points P_1_ to P_6_, according to Figure 8—Equation (1).

(1) $$q_{i\;\max}=q_{i\;\left(+\right)}-q_{i\;\left(-\right)}\;\;\;\mathrm{for}i=1..6\;\;\;\;\;\left[\frac{m}{s^{2}}\right]$$

On this basis, it is possible to assess the displacements of the selected points of the tested hollow block as a result of the mechanical wave propagation, i.e., as a result of the vibration transmission [81]. The measure of damping is the relative mean damping values *w_tm_*, determined according to Equation (2).

(2) $$w_{tm}=\left(1-\frac{q_{m}}{q_{m1}}\right)\cdot 100\;\;\;\left[\%\right]$$

where *q_m_* is the average level of vibration obtained after integrating the signal function *q(t)*, obtained in the time interval from the beginning to the end of the measurement, respectively, for points P_2_ to P_6_ of the back wall of the hollow block, according to Figure 8b. For each of the above measurement points, the value of the average vibration level was determined, and the arithmetic mean was calculated from these values, thus obtaining the average vibration level, denoted as q_m_ in Equation (2), whereas *q_m1_* is the average level of the vibration, obtained in the time interval from the beginning to the end of the measurement, for point P_1_ of the front wall of the hollow block, according to Figure 8a.

To determine the proportional relationship with the energy carried by the evoked signal, the root mean square level RMS is calculated according to Equation (3) [81].

(3) $$q_{s}=\sqrt{\frac{1}{t_{2-}t_{1\;}}\int_{t_{1}}^{t_{2}}{\left(q\left(t\right)-q_{m}\right)}^{2}\mathrm{dt}}\;\;\;\left[\frac{m}{s^{2}}\right]$$

where *t_1_* and *t_2_* are the start and end time of the measurement, respectively, and the *q(t)* signal function and *q_m_* are the mean values of the vibrations obtained after integrating the signal function.

The calculation of the relative mean damping as a root mean square (RMS) value of the vibration for w_ts_ blocks shows a proportional relationship with the energy transformed by the signal. Root mean square takes into account the temporal history of the signal waveform and the amplitude [80,82]. Calculations for a series of three Alpha blocks and a series of three APS new-type hollow blocks were performed in accordance with Equation (4):

(4) $$w_{ts}=\left(1-\frac{q_{s}}{q_{s1}}\right)\cdot 100\;\;\;\left[\%\right]$$

where *q_s_* and *q_s1_* are the values of the root mean square level RMS, for points P_2_ to P_6_ of the back wall of the hollow block and point P_1_ of the front wall of the hollow block, respectively, according to Figure 8.

### 5.1. Description of the Stand for Measuring the Effectiveness of Damping Hollow Blocks

**Figure 9:**
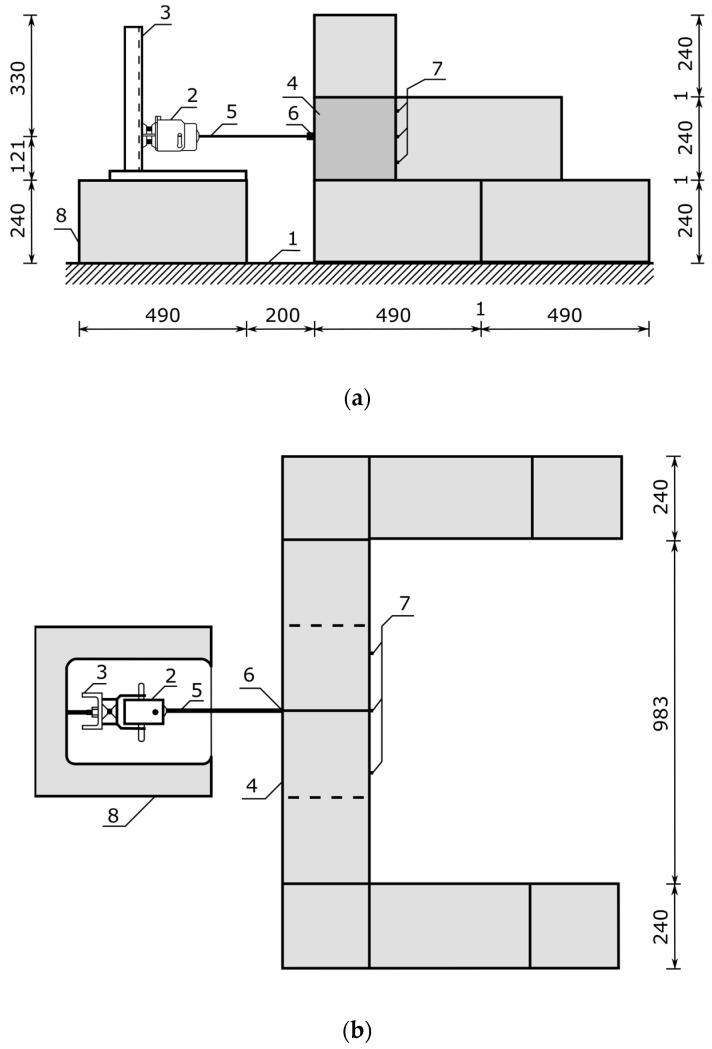
Test stand for measuring the efficiency of block suppression: (**a**) view; (**b**) top view. 1—floor, 2—K2007E01 force exciter, 3—measuring stand, 4—concrete hollow block, 5—M6 rod fixing the force sensor, 6—2311-1 force sensor and P_1_ accelerometer, 7—accelerometers marked in the test with numbers from P_2_ to P_6_, and 8—brick pedestal. Dimensions are in mm.

A test stand was built to study the propagation of mechanical vibrations through concrete wall blocks according to Figure 9. The block wall was placed on the floor by connecting it to the ground with cement mortar. For the tests, three three-row walls were built using the Alpha and APS blocks, respectively. The extreme blocks in the first and second rows of each wall were positioned in a direction perpendicular to the axis of the wall (Figure 9b), thus creating additional bracing for the wall hollow block tested, which was located in the second row in the center of the erected wall (Figure 9a).

An electromagnetic exciter K2007E01 (Manufactured: The Modal Shop, Inc., Cincinnati, OH, USA) with an influence range for a sinusoidal signal with a force of 31 N and a maximum frequency of 9 kHz was placed in a measuring tripod (Figure 9, point 3) and set on two blocks fixed to the ground level with the test block embedded in the wall (Figure 9, point 8). A threaded M6 rod made of nylon was attached to the head of the exciter (Figure 9, point 5), with a force sensor placed at the other end (Figure 9, point 6). The excitation force was applied at point P_1_, i.e., at the geometric center of the front wall of the block, through a 288D01 PCB PIEZOTRONIC force sensor (Manufactured: PCB Piezotronics, Depew, NY, USA) with a frequency range of up to 5 kHz and a maximum measured force of 2224 N (Figure 9, point 6, and Figure 8a).

Accelerometers were attached to each test block studied, according to Figure 8. Six accelerometers were placed on each test block, one on the front wall at point P_1_ and five on the back wall at points P_2_ to P_6_. The first accelerometer, labelled P_1_, was located at the center of the front wall of the hollow block, directly at the point of the force application (Figure 8a). The remaining accelerometers, P_2_ to P_6_, were placed on the back wall of the hollow block, as shown in Figure 8b. The positions of the accelerometers were adopted in the same way for all the tested Alpha and APS hollow wall blocks.

### 5.2. Testing with a Modal Hammer and Discussion of the Results

**Figure 10:**
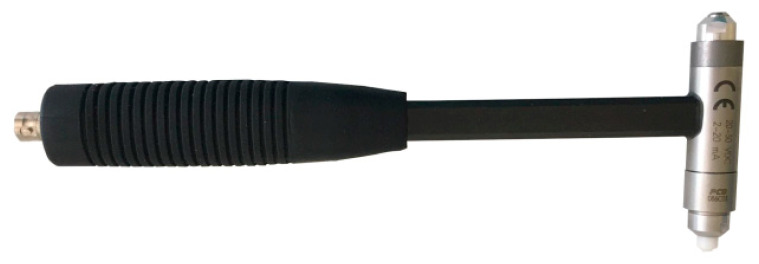
Modal hammer PCB model 086C03 used for testing.

The hollow concrete wall blocks, i.e., the Alpha comparative hollow block and the new APS hollow block, were subjected to the modal hammer test. A PCB modal hammer, model 086C03 PCB Piezotronics (manufactured: PCB Piezotronics, Depew, NY, USA), was used, with a transducer-readable frequency range of up to 8000 Hz and a force amplitude range of 2200 N (Figure 10).

**Figure 11:**
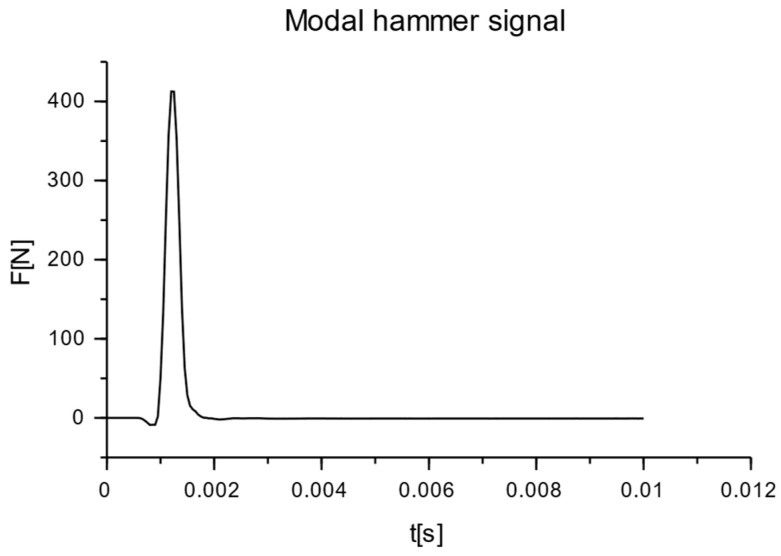
Impulse distribution of excitation force F in time t when hit with a PCB 086C03 modal hammer on a hollow concrete block.

The nature and distribution of the excitation force pulse F over time, when hitting a concrete wall block with a PCB 086C03 modal hammer, is shown in Figure 11. A dedicated plate was selected for the modal hammer, with the goal of obtaining a signal that allowed for the determination of a clear pulse distribution profile. The impact with the modal hammer was repeated until the maximum pulse force F was obtained, ranging from 400 to 415 N. Three trials were thus determined for each series of blocks, in which the discrepancy in the signals was less than 1%. Acceleration values were read from the six accelerometers numbered from P_1_ to P_6_, arranged according to the diagram shown in Figure 8. The results were read using the Sirius DEWESoft’X3 program (manufactured: Dewesoft, Trbovlje, Slovenia).

As a result of the modal hammer test, acceleration amplitudes were recorded for the individual measurement points located on the walls of the Alpha and APS blocks, in accordance with Figure 8. The test was conducted for audible frequencies of up to 20,000 Hz. The graphs are presented for frequencies of up to 4000 Hz. No measurable accelerations were observed above this frequency.

## 6. Test Results for Alpha and APS Hollow Blocks

### 6.1. Assessment of the Efficiency of Hollow Block Damping

The damping effectiveness of the new type of APS concrete wall block compared to the Alpha reference block was tested in the frequency range from 8 Hz to 5000 Hz. The test was carried out on a test stand, as shown in Figure 9, for the selected sixteen frequencies, which were, respectively, 8, 16, 32, 64, 128, 256, 512, 1000, 1500, 2000, 2500, 3000, 3500, 4000, 4500, and 5000 Hz. A sinusoidal waveform of the vibration was forced, and the signal from the accelerometers was recorded at a sampling rate of 200 kHz, using Siemens LMS TestLab software. Three Alpha hollow concrete blocks and three APS blocks were tested. Using Formulas (1) and (2), the peak-to-peak acceleration values, RMS values, and relative mean damping values were calculated $w_{tm}$ for each frequency.

**Figure 12:**
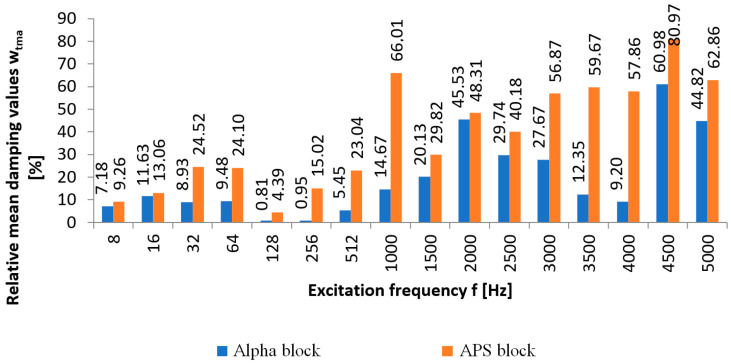
Relative mean damping values $w_{tma}$ [%] between point P_1_—front wall and points P_2_ to P_6_—rear wall, based on the arithmetic mean of measurements for Alpha and APS blocks, in the excitation frequency range of 8 ÷ 5000 Hz.

Based on the arithmetic mean, the relative mean damping values were calculated $w_{tma}$ at points P_2_ to P_6_. For the selected sixteen considered frequencies, the damping values for the Alpha and APS blocks were calculated. A comparison based on the arithmetic mean of the values $w_{tma}$ obtained for the measurement points P_2_ to P_6_ for the relative mean damping values $w_{tma}$ in the excitation frequency range of 8 ÷ 5000 Hz for the Alpha and APS blocks is shown in Figure 12.

The comparison of the relative mean damping values of the new type of APS block with those of the Alpha block revealed that only for the frequencies of 8 Hz, 16 Hz, 128 Hz, and 2000 Hz was the difference small, up to 3.58%. For the other frequencies, a comparison of the relative mean damping values showed a higher efficiency of the APS block, which ranged from 10% to 51%, as shown in Figure 12. An analysis of the relative mean damping values based on the arithmetic mean of the measurements for points P_2_ to P_6_ $w_{tma}$ revealed that the Alpha block, for each excitation frequency studied, had lower damping values than those of the APS. The Alpha hollow block achieved high damping values for the frequencies of 2000 Hz, 4500 Hz, and 5000 Hz and they were, respectively, 45%, 60%, and 44%, but were still lower than the damping for the APS hollow blocks.

In other cases, the relative mean damping did not exceed 30%. At low frequencies of 8 to 256 Hz, the concrete blocks of the new type achieved relative mean damping values greater than several to ca. 30%, and for higher frequencies, the damping definitely increased and reached values of up to 80% in favor of the APS blocks. Based on the comparison of the damping of the new type of APS blocks with the Alpha blocks, it can be concluded that only for the frequency of 2000 Hz was the damping of the new type of hollow blocks comparable with the Alpha blocks and within 45–55%. In other cases, the new type of APS hollow blocks was significantly better at reducing mechanical vibrations.

**Figure 13:**
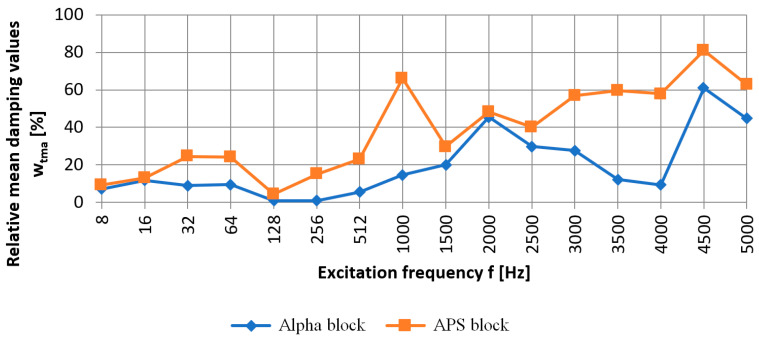
Relative mean RMS damping $w_{tsa}$ [%] between point P_1_—front wall and points P_2_ to P_6_—rear wall based on the arithmetic mean of measurements for Alpha and APS blocks in the frequency range of 8 ÷ 5000 Hz.

Based on Equation (4), the relative mean RMS damping $w_{tsa}$ in the excitation frequency range of 8 ÷ 5000 Hz for the Alpha and APS hollow blocks was calculated for the arithmetic mean of the measurements at points P_2_ to P_6_ located on the back wall of the tested hollow blocks. The results are shown in Figure 13.

The comparison of the relative mean RMS damping $w_{tsa}$ in the band of the analyzed frequencies showed that the new APS hollow wall block was more effective in reducing mechanical impacts than the Alpha block. Only for the frequencies of 128 Hz and 256 Hz, both considered hollow wall blocks, was similar relative mean RMS damping $w_{tsa}$ shown.

### 6.2. Test Results of Free Vibration

**Figure 14:**
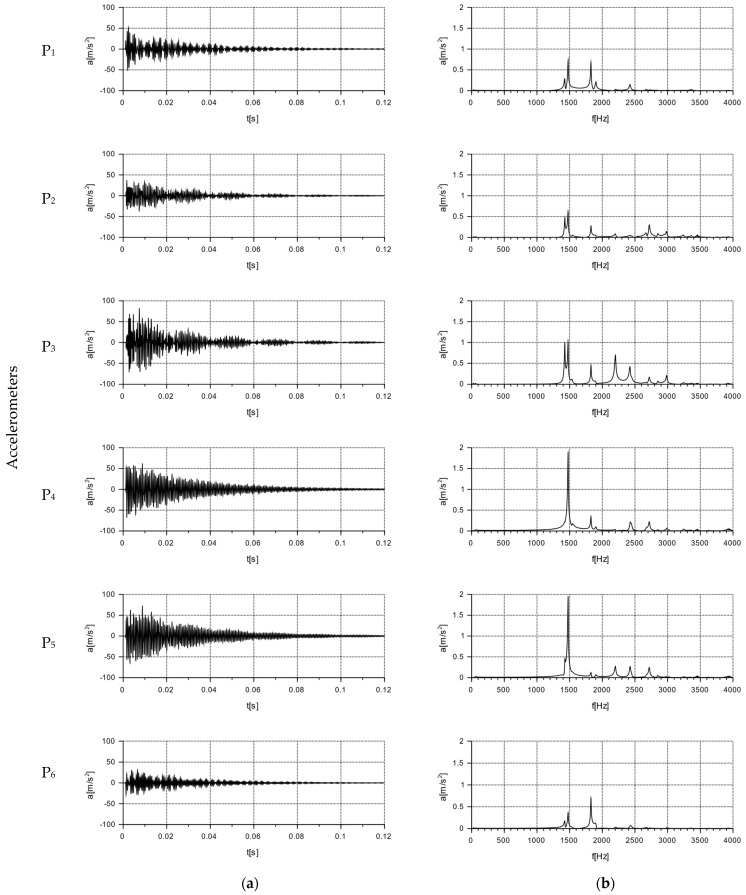
Graphs of free vibrations for the Alpha block as a result of the impact with a modal hammer with a force of 412 N for accelerometers set at the measurement points, according to the diagram in Figure 8 and Figure 16. Acceleration value: (**a**) in the time domain; (**b**) in the frequency domain (spectrum).

**Figure 15:**
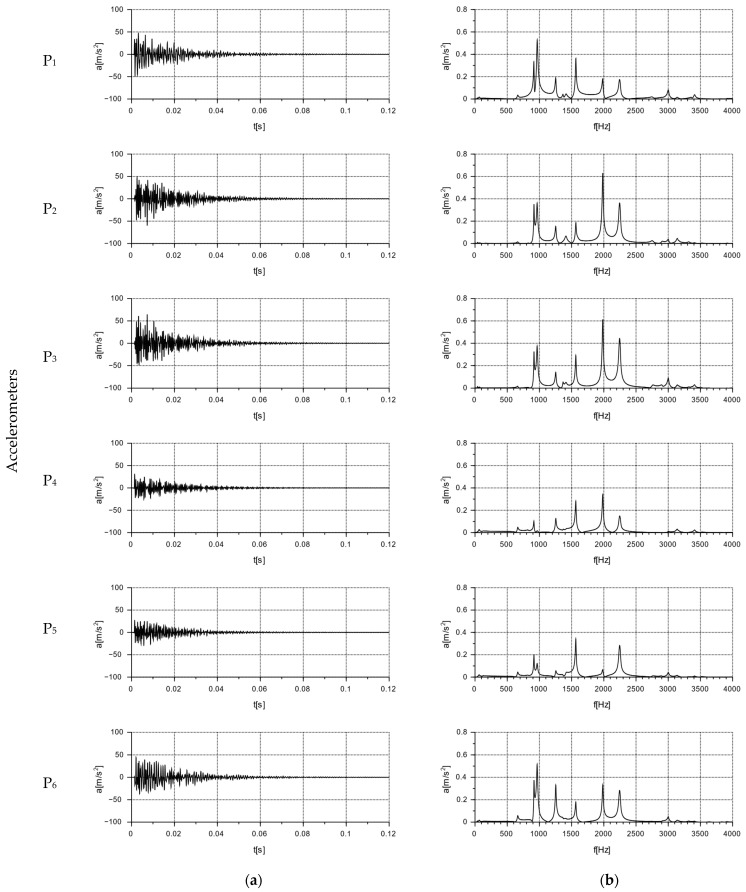
Graphs of free vibrations for the APS block as a result of the impact with a modal hammer with a force of 400 N for accelerometers set at the measurement points, according to the diagram in Figure 8 and Figure 16. Acceleration value: (**a**) in the time domain; (**b**) in the frequency domain (spectrum).

**Figure 16:**
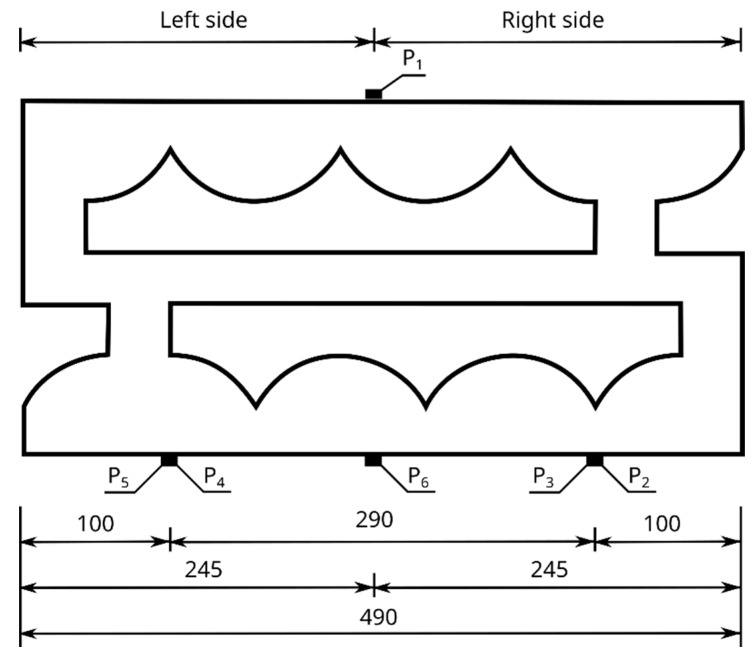
View of the arrangement of accelerometers P_2_ to P_6_ in the horizontal plane.

The results for the tested Alpha and APS blocks as a result of the modal hammer impacts are shown in Figure 14 and Figure 15, in the form of an amplitude acceleration spectrum of amplitude in the time domain and a spectrum of amplitude in the frequency domain. The application of the force causing the vibration occurred at a distance of 1 cm from the accelerometer P_1_ (Figure 8). The characteristics of the applied force are shown in Figure 8. The measuring points P_2_ and P_3_ were set on the right side of the back wall of the hollow blocks at the narrow rib. The measuring points P_4_ and P_5_ were set on the left side of the back wall of the hollow blocks, close to the wider rib. The arrangement of the accelerometers at the selected points is shown in Figure 8 and Figure 16.

As a result of the impact with the modal hammer, the comparison Alpha hollow block was excited with a force of 412 N. The vibration extinction time was about 0.1 s. The amplitude of the acceleration in the frequency domain is shown in the second column of Figure 14, which demonstrated a similar character (regardless of the location of the accelerometer) in the frequency range of 0 ÷ 4000 Hz. An increase in the vibration amplitudes at similar frequencies was observed for all the selected points: about 1424 Hz, 1825 Hz, 2428 Hz, and 2715 Hz. A comparison of the data from the accelerometers placed on the left side (P_4_, P_5_) and the right side (P_2_, P_3_) of the rear wall of the block (Figure 16) revealed the highest acceleration amplitudes on accelerometers P_4_ and P_5_ placed on the rib for a frequency of 1478.63 Hz. An analysis of the data from the P_1_ and P_6_ accelerometers at the centers of the front and back panels found identical frequencies in two peaks, a lower one at around 1475 Hz, and a higher one at around 1827 Hz. For a frequency of 1475 Hz, with a maximum input acceleration amplitude at P_1_ of 0.7 m/s^2^, the value of the acceleration at P_6_ on the back wall was half of the input value, at 0.348 m/s^2^.

The test was carried out for a new type of concrete wall hollow block (APS) induced with a force of 400 N using a modal hammer. The vibration extinction time was ca. 0.08 s. The acceleration amplitude in the frequency domain is shown in the second column of Figure 15. The maximum acceleration value was up to 50 m/s^2^, with a frequency range of 0 ÷ 4000 Hz. A comparison of the data from accelerometers P_1_ and P_6_ placed at the geometric centers of the front and rear walls allowed for the determination of the maximum acceleration amplitude at the input at P_1_ at the level of 0.65 m/s^2^ and at the output at P_6_ at the level of 0.516 m/s^2^. At higher frequencies, there were also decreases in the acceleration amplitudes at point P_6_ compared to point P_1_. Comparing the data of the accelerometers placed on the left side (P_4_, P_5_) and on the right side (P_2_, P_3_) of the rear wall of the hollow block (Figure 16), lower acceleration amplitudes were observed on the accelerometers P_4_ and P_5_ located on the rib for the frequencies of 915 Hz, 1563 Hz, 1892 Hz, 2248 Hz, and 3001 Hz.

A free vibration plot was obtained for both types of blocks as a result of using a modal hammer with a force of 400 N. The vibration extinction time for the Alpha block was about 0.1 s, while that for the new APS block was about 0.08 s. These values were read assuming that the vibration extinction cut-off point occurred at a = 2 m/s^2^—see Table 8. A comparison of the new concrete blocks with the Alpha block revealed that the vibration extinction time was reduced by 25% compared to the Alpha block.

Furthermore, a comparison of the readings from the accelerometers placed at measurement points P_1_ to P_6_ for the new type of APS blocks showed that all the amplitude readings were lower than those from the accelerometers placed on the Alpha block. This demonstrated the effectiveness of the solution presented.

## 7. Testing of the Damping Effectiveness of APS Hollow Blocks

### 7.1. Discussion of the Nature of the Research

**Figure 17:**
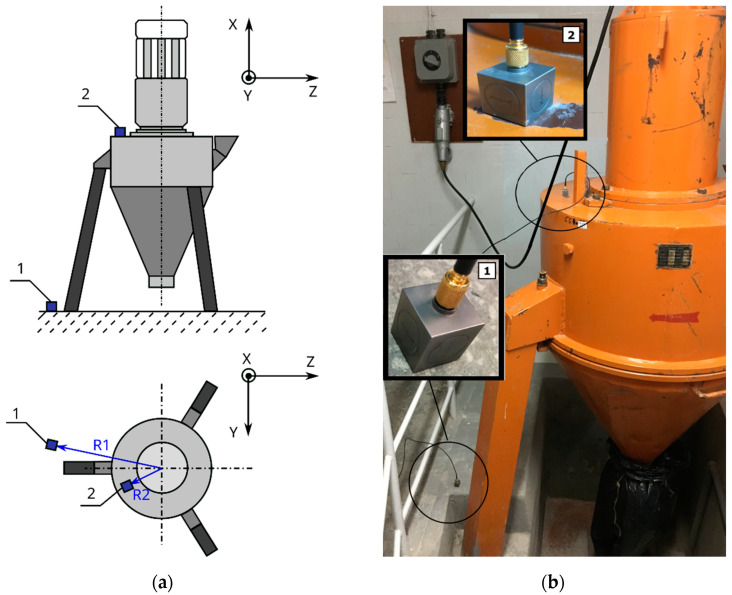
Impact mill with pneumatic circuit, type M-400: (**a**) scheme of the device with assigned setting directions of accelerometers and distances from the mill axis R1 = 75 cm, R2 = 40 cm; (**b**) photo of the device; 1—location of the accelerometer on the concrete floor, and 2—location of the accelerometer on the mill body.

An example of the analysis of the effectiveness of the APS block damping was performed for conditions occurring at the Faculty of Civil Engineering at the Częstochowa University of Technology, where the source of high-intensity vibrations is an M-400 pneumatic impact mill with a capacity of 11 kW, which is designed for grinding bulk materials. It is driven by an SKF-160M-2A motor (Manufactured: Fumo-Ostrzeszów, Ostrzeszów, Poland) and powered by 400 V, with a rated rotor speed of 3000 rpm. To determine its dynamic impact on the external environment, measurements were taken while the mill was operated. During the vibration measurements, the device was loaded with 20 kg of loose material in the form of fine aggregate with a fraction of 2÷8 mm. Due to the positioning of the impact mill directly on the concrete floor in the laboratory, measurements were taken at two points, (1) on the floor and (2) on the body of the mill, in accordance with Figure 17. The test was performed using accelerometers, which indicated the acceleration values over time in three directions, x, y, and z; the results were read using the Simens LMS TestLab 17 program.

For the test results, Fourier FFT transform was performed to transform the signal from the time domain to the frequency domain. This made it possible to present a frequency representation of the signal, and the signal spectrum contained information about the “signal frequency content”. The resulting transformation can be interpreted as the determination of the measure of the correlation for individual harmonic functions, i.e., checking “how much” of a particular “frequency” is contained in the signal.

### 7.2. Determination of the Nature of Mechanical Vibrations

**Figure 18:**
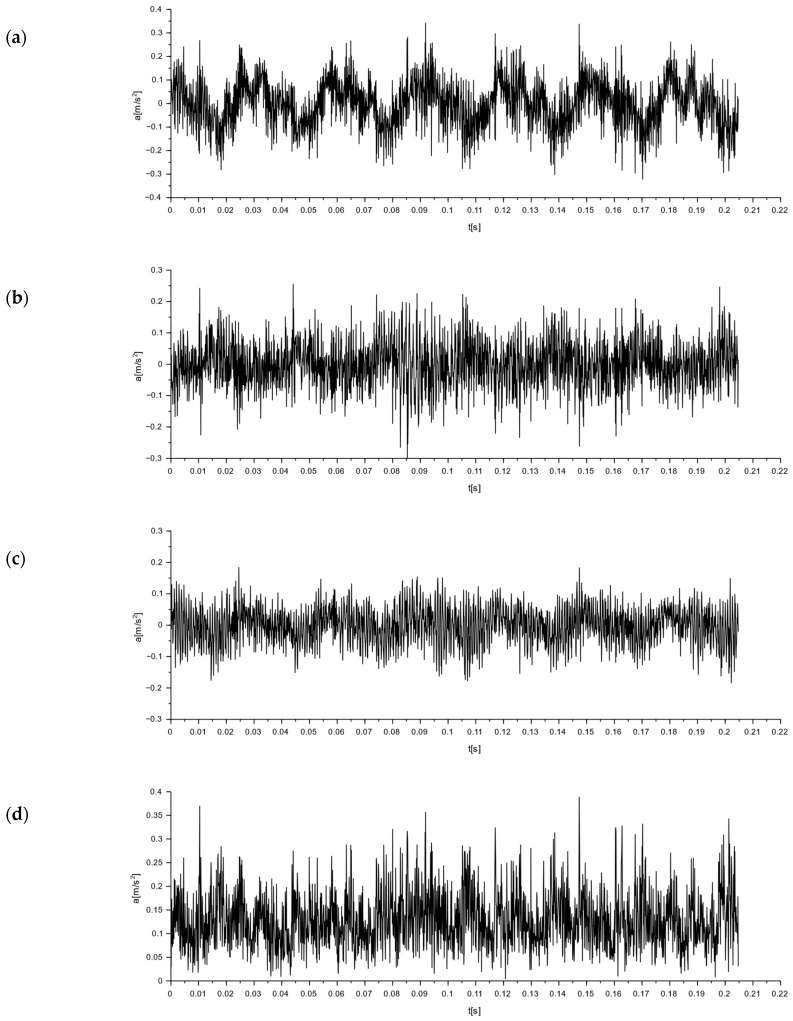
Graphs of vibrations induced by an impact mill with a pneumatic circuit, type M-400. Signal from the accelerometer placed on the floor next to the mill (Figure 17, point 1): (**a**) for x-direction; (**b**) for y-direction; (**c**) for z-direction; and (**d**) for the resultant acceleration.

**Figure 19:**
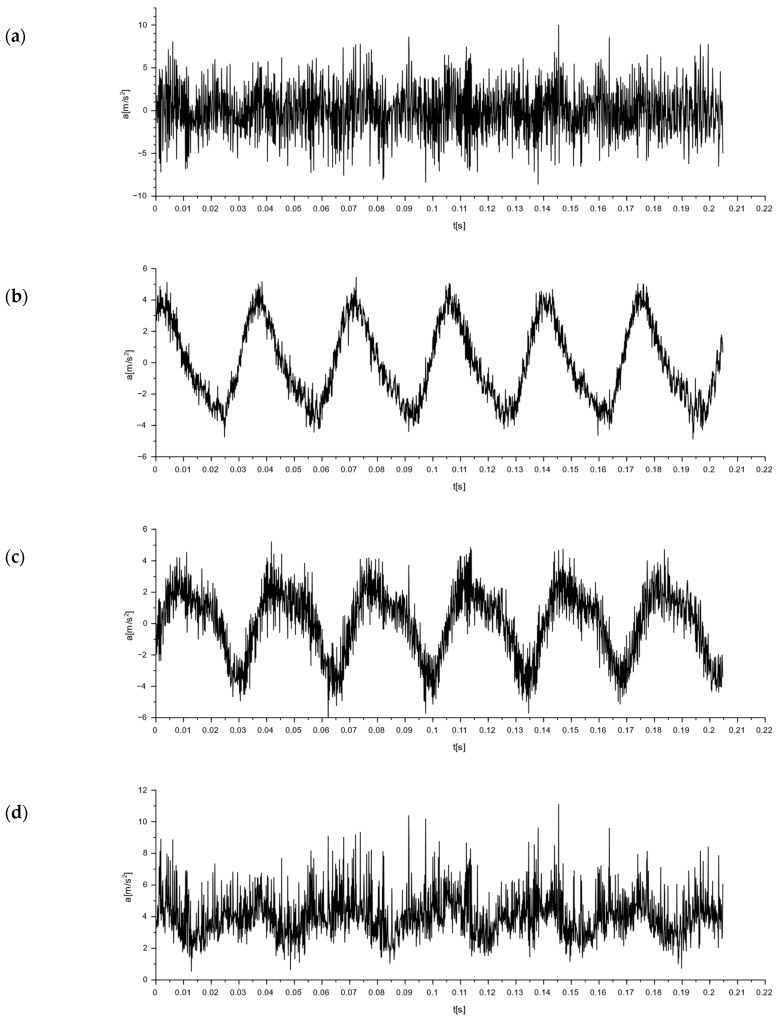
Graphs of vibrations induced by an impact mill with a pneumatic circuit, type M-400. Signal from the accelerometer set on the mill body (Figure 17, point 2): (**a**) for x-direction; (**b**) for y-direction; (**c**) for z-direction; and(**d**) for the resultant acceleration.

To determine the nature of the mechanical vibrations of the M-400 impact mill, measurements were made with a triaxial accelerometer. The signal values recorded on the floor at the mill are shown in Figure 18. In Figure 18d, the resultant for the measurements taken is additionally determined. Acceleration graphs for each axis from the sensor set on the mill body are shown in Figure 19. The resultant from the measurements taken is also determined here (see Figure 19d).

By analyzing the readings from a sensor placed on the floor at the mill, the vibrations with the highest amplitude were obtained in the x-direction, and they were 0.32 m/s^2^ (Figure 18). These were sinusoidal vibrations, which are a consequence of the operating characteristics of the analyzed device. The y- and z-directions also showed sinusoidal oscillations, with their values being an order of magnitude smaller than those measured in the x-direction.

The readings from a sensor placed on the body of the mill indicated vibrations with the highest amplitude also in the x-direction. Their value was 9.97 m/s^2^ (Figure 19). These were sinusoidal vibrations. In the y- and z-directions, half the vibration was obtained, but with the same sinusoidal waveform.

### 7.3. Simulation of Damping of Selected Signals

**Figure 20:**
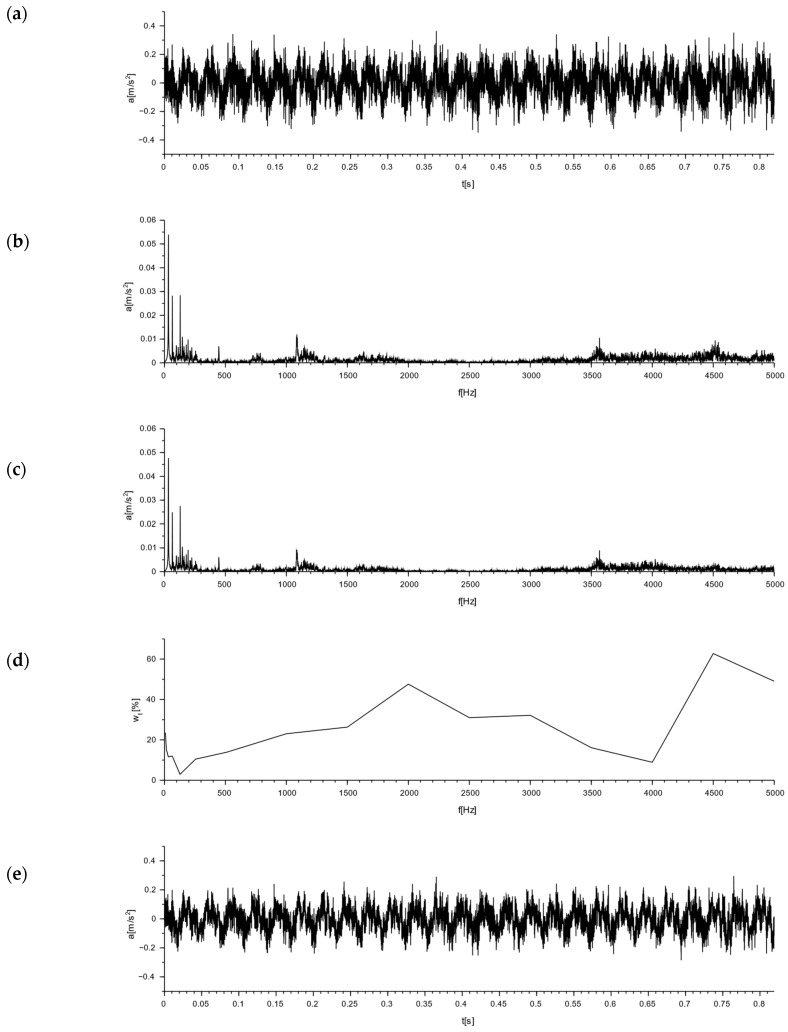
Simulation of signal damping from the work of an impact mill with a pneumatic circuit, type M-400, read from a sensor placed on the floor next to the mill for the Alpha block: (**a**) acceleration plot—time domain signal exciting Alpha block by M-400 mill; (**b**) the frequency domain signal transformed from the signal of subsection (**a**); (**c**) the simulated signal in the frequency domain damped by Alpha block, obtained from the plot (**b**) by taking into account the damping coefficients from the plot (**d**); (**d**) plot of damping coefficients vs. frequency; and (**e**) the simulated acceleration after Alpha block damping for the M-400 mill excitation signal, the signal in the time domain obtained from the transformation of the plot from subsection (**c**).

**Figure 21:**
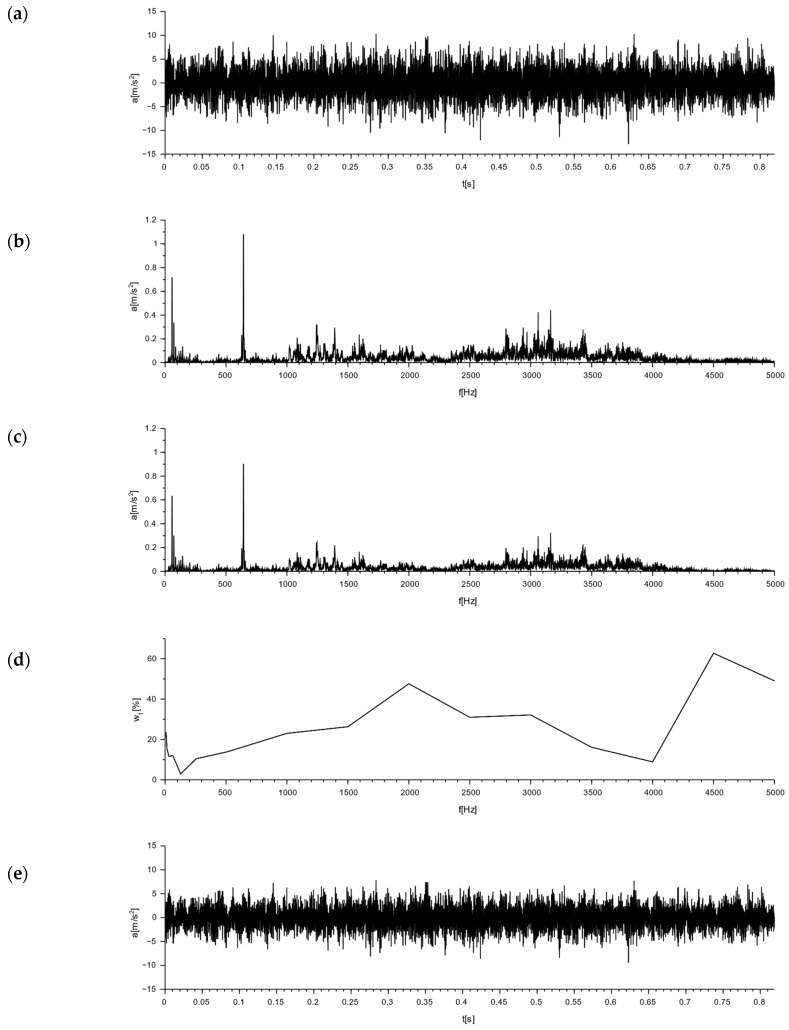
Simulation of signal damping from the work of an impact mill with a pneumatic circuit, type M-400, read from the sensor set on the mill body for the Alpha block: (**a**) acceleration plot—time domain signal exciting Alpha block by M-400 mill; (**b**) the frequency domain signal transformed from the signal of subsection (**a**); (**c**) the simulated signal in the frequency domain damped by Alpha block, obtained from the plot (**b**) by taking into account the damping coefficients from the plot (**d**); (**d**) plot of damping coefficients vs. frequency; and (**e**) the simulated acceleration after Alpha block damping for the M-400 mill excitation signal, the signal in the time domain obtained from the transformation of the plot from subsection (**c**).

**Figure 22:**
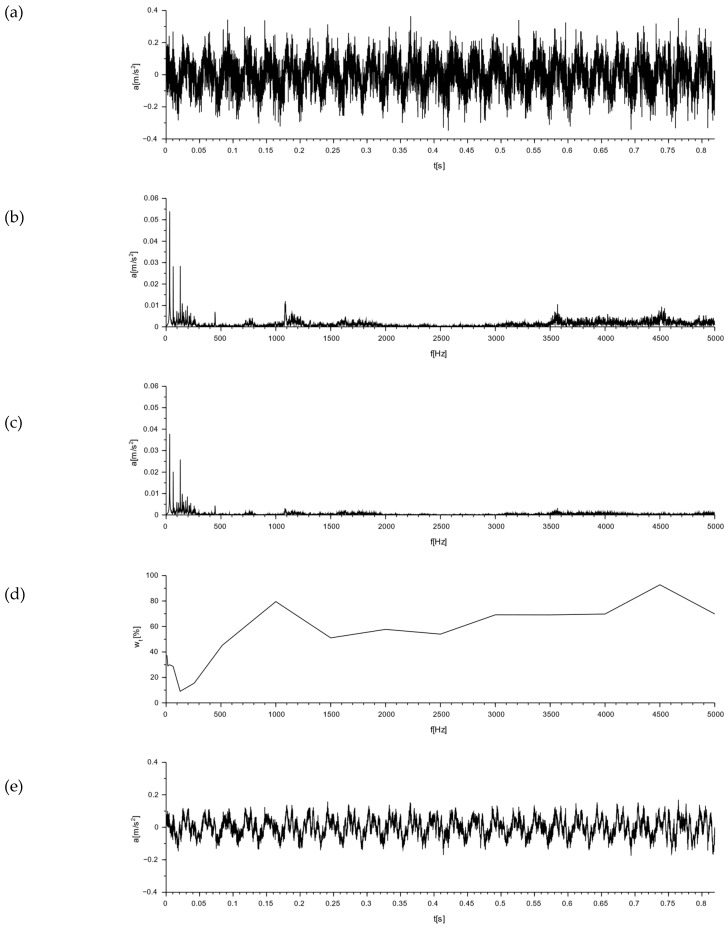
Simulation of signal damping from the work of an impact mill with a pneumatic circuit, type M-400, read from a sensor placed on the floor next to the mill for the APS block: (**a**) acceleration plot—time domain signal exciting APS block by M-400 mill; (**b**) the frequency domain signal transformed from the signal of subsection (**a**); (**c**) the simulated signal in the frequency domain damped by APS block, obtained from the plot (**b**) by taking into account the damping coefficients from the plot (**d**); (**d**) plot of damping coefficients vs. frequency; and (**e**) the simulated acceleration after APS block damping for the M-400 mill excitation signal, the signal in the time domain obtained from the transformation of the plot from subsection (**c**).

**Figure 23:**
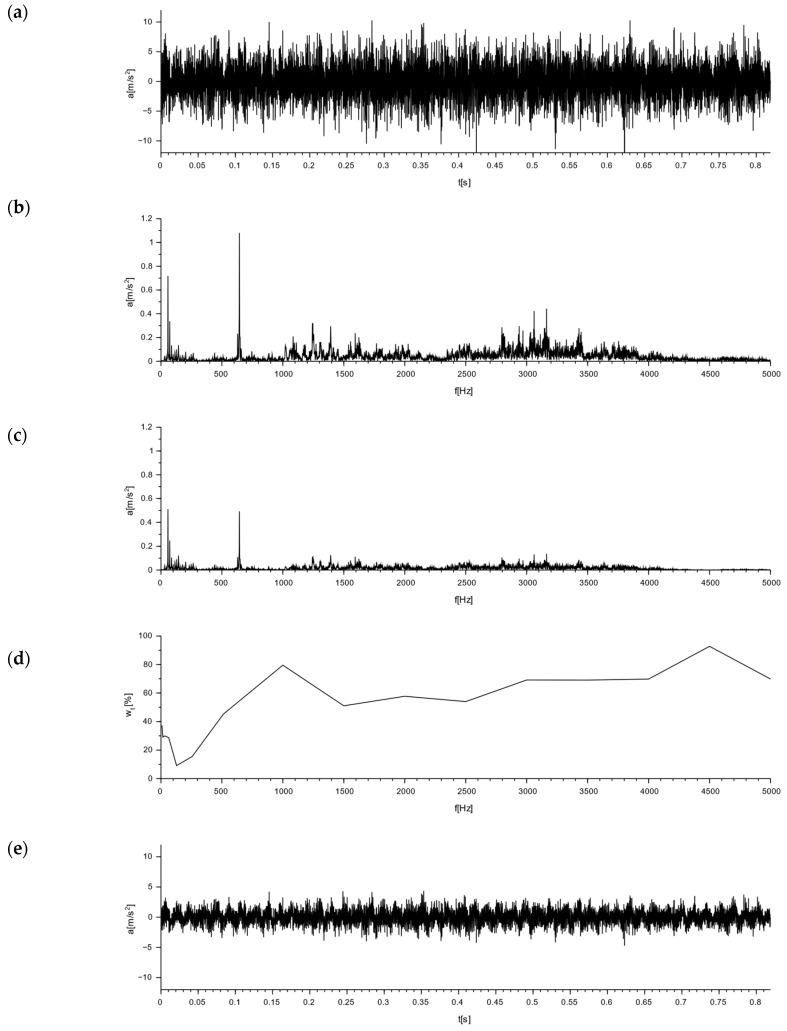
Simulation of signal damping from the work of an impact mill with a pneumatic circuit, type M-400, read from the sensor set on the mill body for the APS block: (**a**) acceleration plot—time domain signal exciting APS block by M-400 mill; (**b**) the frequency domain signal transformed from the signal of subsection (**a**); (**c**) the simulated signal in the frequency domain damped by APS block, obtained from the plot (**b**) by taking into account the damping coefficients from the plot (**d**); (**d**) plot of damping coefficients vs. frequency; and (**e**) the simulated acceleration after APS block damping for the M-400 mill excitation signal, the signal in the time domain obtained from the transformation of the plot from subsection (**c**).

A determination of the nature of the interactions for the M-400 impact mill made it possible to analyze the damping efficiency of the Alpha and APS blocks. This was obtained via a simulation analysis of the damping of the vibrations forced by the operation of the M-400 impact mill for the tested blocks. For the Alpha block, signal damping simulations are shown in Figure 20 and Figure 21, whereas for the APS block, they are shown in in Figure 22 and Figure 23.

### 7.4. Discussion of the Results

Figure 20, Figure 21, Figure 22 and Figure 23 sequentially show, in subsections: (a) an acceleration plot—the time domain signal exciting the block, (b) the frequency domain signal transformed from the signal of the subsection (a), (c) the simulated signal in the frequency domain damped by the block, obtained from plot (b) by taking into account the damping coefficients from plot (d), (d) a plot of the damping coefficients vs. frequency, and (e) the simulated acceleration after the block damping for the excitation signal, and the signal in the time domain obtained from the transformation of the plot from subsection (c).

Based on the tests performed, the relative mean damping values $w_{tma}$ were calculated for the frequency range from 8 to 5000 Hz and the relative mean RMS damping $w_{tsa}$ for the Alpha and APS blocks, taking into account the signal generated by an M-400 pneumatic impact mill in two variants: 1—with a measurement on the floor next to the mill and 2—with a measurement on the mill body. A signal analysis was performed for three directions, x, y, and z, and their resultants.

**Figure 24:**
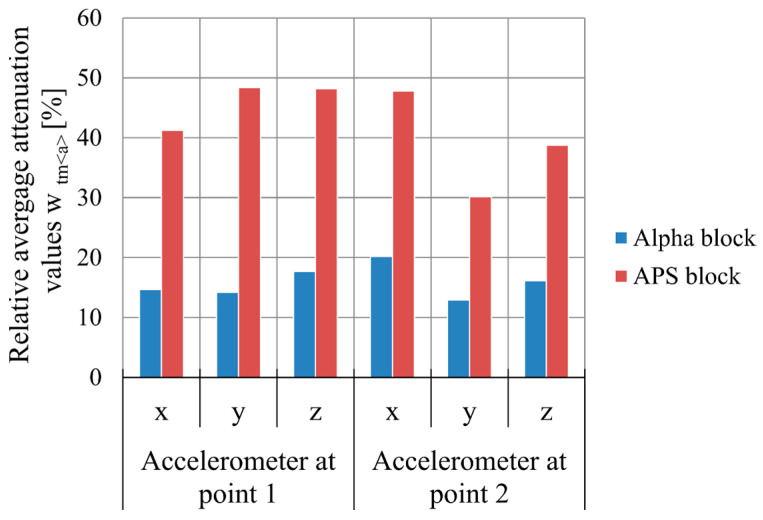
Relative mean damping values $w_{tma}$ [%] of the mill with pneumatic circuit, type M-400, for Alpha and APS hollow concrete blocks in the x-, y-, and z-directions.

**Figure 25:**
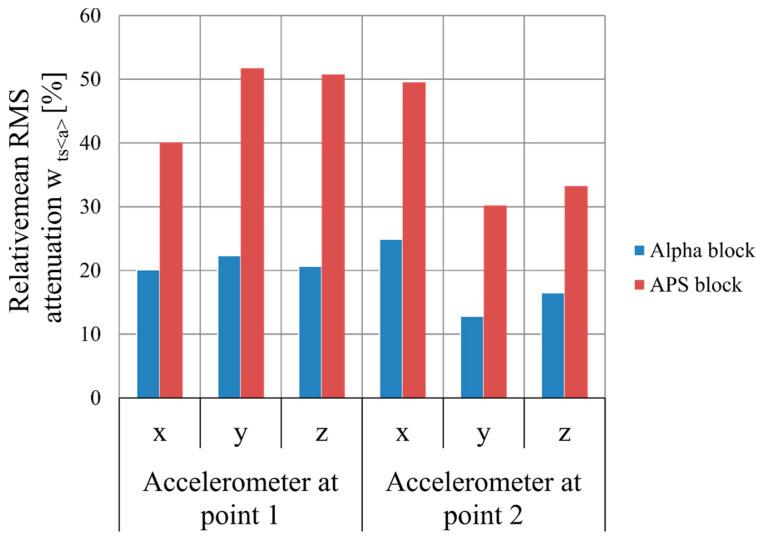
Relative mean damping of the RMS $w_{tsa}$ [%] of the mill with pneumatic circuit, type M-400, for Alpha and APS concrete blocks in the x-, y-, and z-directions.

A comparison of the relative mean damping values $w_{tma}$ for the tested hollow blocks is shown in Figure 24, and for the relative mean RMS damping $w_{tsa}$, the comparison is shown in Figure 25.

A comparison of the simulated damping for the signal measured on the floor at the leg of the M-400 pneumatic impact mill during its operation, at a distance of 75 cm from its axis (point 1 in Figure 17), shows that the relative mean RMS damping $w_{tsa}\;$for the Alpha block was 23.11%, while that for the APS block was 39.98%. On the mill body, at a distance of 40 cm from its axis (point 2 in Figure 17), it was found that the relative mean RMS damping $w_{tsa}$ for the Alpha block was 14.92%, while for the APS block, it was at a level more than twice as high, at 33.60%. In this case, more than a 100% improvement in the vibration reduction efficiency was achieved.

For the signal generated by the operation of the M-400 pneumatic impact mill, read from a sensor set at the mill on the floor, the APS block absorbed 16.87% more signal compared to the Alpha block, while for the signal read from a sensor set on the mill body, the APS block absorbed 18.68% more signal compared to the Alpha block. A simulation of the damping of the impact mill signal showed that, for both signals analyzed, the damping of the APS blocks was significantly higher compared to the Alpha comparison block.

## 8. Conclusions and Summary of the Research

The paper presented an innovative APS concrete wall block and provided technical data on this new type of block, the percentages of the SBR rubber granules and PET flakes used in the modified concrete, and the composition of the concrete modified with this mixture of recycled additives (SBR rubber granules and PET flakes) per 1 m^3^ volume of concrete mix.

To demonstrate the effectiveness of the solution in reducing mechanical vibrations, the effect of the interaction of different frequencies of the mechanical wave on reducing these vibrations was evaluated for comparison blocks (Alpha) and innovative wall blocks (APS). The test was performed on a test stand construed for dynamic measurements following the propagation of a mechanical wave, thus determining the damping efficiency of the blocks. The study was conducted for sixteen frequencies: 8, 16, 32, 64, 128, 256, 512, 1000, 1500, 2000, 2500, 3000, 3500, 4000, 4500, and 5000 Hz.

The comparison of the relative mean damping values of the APS block with those of the Alpha block showed that, for low frequencies of 8 Hz, 16 Hz, and 128 Hz and frequencies of 2000 Hz, respectively, the difference in damping was up to 3.58% in favor of the new type of hollow block (APS). For the other frequencies tested, the comparison of the damping of the APS block with the Alpha block ranged from 10% to 51%, confirming its usefulness in reducing mechanical vibrations. The analysis of the relative mean damping values revealed that the Alpha block, for each excitation frequency tested, was less effective at damping the signal than the new type (APS). Significant damping was obtained for the Alpha block for frequencies of 2000, 4500, and 5000 Hz. They were over 45%, 60%, and 44%, respectively. For the other frequencies tested, the Alpha block showed significantly lower damping values, which did not exceed 30% compared to the new APS block. The comparison Alpha block and the APS block were also subjected to a modal hammer test. This allowed for an analysis of the nature of the free vibration for each block tested. Based on the acceleration amplitudes read from the accelerometers placed on the front and rear walls of the tested blocks, the new APS block was found to have a 25% shorter impact pulse extinction time compared to the Alpha block.

A simulation of the damping of the vibrations forced by the M-400 pneumatic impact mill was also carried out for the tested Alpha and APS blocks. Based on the resultant readings from the accelerometers located on the body of the impact mill and the floor at the mill, the nature of the interactions was determined, and an analysis of the damping efficiency of the test blocks was carried out. For the signal measured on the mill body, the relative mean RMS damping $w_{tsa}\;$was evaluated, which was 14.92% for the Alpha block and 33.60% for the APS block. On the floor directly at the mill, the value of $w_{tsa}\;$for the Alpha block was 23.11%, while for the APS block it was 39.98%. This demonstrated the greater effectiveness of the new type of concrete wall block (APS) in reducing mechanical vibrations compared to the Alpha block. In conclusion, the research on the reduction in mechanical interactions, both in terms of the sixteen frequencies studied and with regard to the effectiveness of the solution studied at damping the vibrations forced by the operation of the M-400 pneumatic impact mill, showed that the new type of APS concrete wall hollow block is an effective alternative to other types of concrete matrix blocks used for structural wall masonry and can significantly improve the comfort of buildings subjected to mechanical vibrations. Furthermore, the developed solution effectively uses recycled materials in the form of SBR rubber granules from, for example, used car tires, and PET flakes from used food packaging, to produce modified concrete for a new type of APS concrete wall hollow block.

The developed APS wall hollow block is an innovative solution protected by Patent No PL 235427 B1 [67]. In order to obtain its effectiveness at reducing the vibrations presented in this study, a purely mechanical approach was needed, resulting from the use of the geometry of the internal space of the hollow block, through the proper design of its through-holes and butt locks to reduce the vibration energy. This required many numerical analyses, which took into account sublime research methods. In the course of the numerical research prior to obtaining the patent protection, a preliminary strength assessment was also performed, which was confirmed at the stage of experimental verification, both in the study of the concrete mix modified with recycling additives and the prototype series of the APS hollow blocks. An additional test confirming the effectiveness of the developed solution, based on a non-traditional approach to the design of concrete hollow blocks, was the simulation of vibrations generated by the M-400 pneumatic impact mill, intended for grinding loose materials. For the developed solution, the reduction in the signal for the APS wall block was greater than that of the comparative Alpha hollow block, by 16.87% from the signal reading on the floor next to the mill and by 18.68% from the signal reading from the mill body, in accordance with Figure 17. In this way, the effectiveness of the vibration reduction in the internal structure of the hollow block was demonstrated, which was also observed at the stage of testing sinusoidal excitations in the range from 8 to 5000 Hz.

To sum up, it can be stated that the APS concrete hollow block discussed in this paper, designed with a concrete mix modified with recycling additives (SBR and PET), not only meets the standard criteria for this type of construction product, but also has an innovative solution for its internal structure, showing a greater efficiency (compared to the Alpha hollow block) in reducing propagating vibrations. This was the most important design intention, which, on the basis of the research cited here, can be considered as meeting the expectations of our work.

## 9. Patents

The authors obtained, in Poland, a patent for the invention: Ażurowy pustak ścienny (Openwork wall hollow brick), Patent No PL 235427 B1, Major, M.; Adamczyk-Królak, I. Czestochowa University of Technology 2020.

## Figures and Tables

**Table 1:** Technical data of the new type of APS block made of modified concrete in accordance with Table 3.

Real dimensions	Length	490 mm/+3−5 mm
	Width	240 mm/+3−5 mm
	Height	240 mm/+3−5 mm
Shape and construction		Group 2 according to EN 1996-1-1 [70]
Item weight		43.8 kg
Compressive strength (perpendicular to the laying surface 490 × 240)		26.7 N/mm^2^
Water absorption		4.9 g/(m^2^s)
Durability (resistance to freeze/thaw)		Frost resistant

**Table 2:** Percentage shares of SBR rubber granulates and PET flakes used in modified concrete for APS blocks.

Additives from Recycled Waste	100 [%]	Modified Concrete Series
		15% by Weight of Cement
Rubber granulate 0 ÷ 1 mm	90	18%
Rubber granulate 0.8 ÷ 2 mm		18%
Rubber granulate 2 ÷ 4 mm		54%
PET flakes	10	10%
Sum of additions	100	100%

**Table 3:** Composition of concrete modified with a mixture of recycling additives: SBR rubber granules and PET flakes of APS blocks per volume of 1 m^3^ of concrete mix.

c/w	w/c	CEM I 32.5R Cement [kg/m^3^]	Water after Correction [l/m^3^]	Washed Sand [kg/m^3^]	Gravel 2 ÷ 8 mm [kg/m^3^]	Stacheplast 202N Superplasticizer in the Amount of 1.2% of the Cement Weight [l/m^3^]	Percentage of Additives in the Mass of Cement [%]	SBR Rubber Granules [kg/m^3^]			PET Flakes [kg/m^3^]
								0 ÷ 1 mm	0.8 ÷ 2 mm	2 ÷ 4 mm	
2.1	0.476	416	193.1	306	1107	4.99	15	11.23	11.23	33.69	6.24

**Table 4:** Results of compressive strength tests after 7, 14, and 28 days for modified concrete used to make APS hollow blocks.

APS Hollow Block	Average Compressive Strength f_cm_ [MPa]			Concrete Strength Class
	After 7 Days	After 14 Days	After 28 Days	
Modified concrete	34.0	37.5	39.8	C25/30

**Table 5:** Physical and chemical properties of SBR rubber granules. Classification according to [72].

Physical and Chemical Properties	SBR Rubber Granules
Form	Granulate
Color	Black
Smell	Mild
Density	350 ÷ 700 kg/m^3^
Solubility	It does not dissolve in water
Ignition point	>350 °C
Ignition temperature	>350 °C
Thermal degradation	>180 °C
A dangerous decay/products	SO_x_, N0_x_, organic hydrocarbons, degradation at temperatures below 800 °C and in conditions of oxygen deficiency—intensive formation of soot

**Table 6:** Physical and chemical properties of polyethylene terephthalate in the form of colored PET flakes [73].

Physical and Chemical Properties	Polyethylene Terephthalate in the Form of Colored PET Flakes
Form	Flakes
Color	Colorful
Intrinsic viscosity	0.74 ± 0.03 dL/g
The size of the flakes	<12 mm
Density	260 ± 50 kg/m^3^
Humidity	<1%
Specific density	Approx. 1.35 g/cm^3^
Dust units	<0.5%
Metal	<100 ppm
Paper	<50 ppm
PVC	<50 ppm
Flakes covered with glue	<1000 ppm

**Table 7:** Technical data of the Alpha block [74].

Dimensions	Length	490 mm/+3−5 mm
	Width	240 mm/+3−5 mm
	Height	240 mm/+3−5 mm
Shape and construction		Group 2 according to EN 1996-1-1 [70]
Item weight		46 kg
Compressive strength (perpendicular to the laying surface 490 × 240)		25 N/mm^2^
Water absorption		5.5 g/(m^2^s)
Durability (resistance to freeze/thaw)		Frost resistant

**Table 8:** Acceleration values for Alpha and APS hollow blocks read from Figure 14 and Figure 15 for 12 time steps from 0.01 s to 0.12 s at point P_6_.

No.	t [s]	a [m/s^2^]	
		Alpha	APS
1.	0.01	36.72	31.03
2.	0.02	26.12	22.27
3.	0.03	18.86	15.11
4.	0.04	14.02	10.21
5.	0.05	10.22	7.09
6.	0.06	7.12	4.81
7.	0.07	5.06	3.84
8.	0.08	3.89	1.95
9.	0.09	2.85	1.44
10.	0.10	1.92	1.12
11.	0.11	1.69	0.87
12.	0.12	1.35	0.75

## Data Availability

Not applicable.

## References

[1] Hasan M., Saidi T., Sarana D. (2022). The strength of hollow concrete block walls, reinforced hollow concrete block beams, and columns. J. King Saud Univ. Eng. Sci..

[2] Papliński A.T., Bloczki Kontra Pustaki (2023). Dom z Betonu Komórkowego czy Ceramiki?. Murator.

[3] Ołdakowska E. (2006). Beton cementowy modyfikowany rozdrobnionymi odpadami gumowymi (Cement concrete modified by fragmented rubber waste). Zesz. Naukowe. Bud..

[4] Duda A., Sobota D. (2017). Badania zużytych opon do wykorzystania w budownictwie (Testing tyre bales from compressed used car tyres for use in construction). Builder.

[5] Ramdani S., Guettala A., Benmalek M.L., Aguiar J.B. (2019). Physical and mechanical performance of concrete made with waste rubber aggregate, glass powder and silica sand powder. J. Build. Eng..

[6] Fraile-Garcia E., Ferreiro-Cabello J., Mendivil-Giro M., San Vicente-Navarro A. (2018). Thermal behaviour of hollow blocks and bricks made of concrete doped with waste tyre rubber. Constr. Build. Mater..

[7] Saikia N., de Brito J. (2014). Mechanical properties and abrasion behaviour of concrete containing shredded PET bottle waste as a partial substitution of natural aggregate. Constr. Build. Mater..

[8] Akcaozoglu S., Atis C.D., Akcaozoglu K. (2010). An investigation on the use of shredded waste PET bottles as aggregate in lightweight concrete. Waste Manag..

[9] Rahmani E., Dehestani M., Beygi M.H., Allahyari H., Nikbin I. (2013). On the mechanical properties of concrete containing waste PET particles. Constr. Build. Mater..

[10] Benosman A.S., Taibi H., Belbachir M., Senhadij Y. (2008). Diffusion of chloride ions in polimer-mortar composities. J. Appl. Polym. Sci..

[11] Qaidi S., Al-Kamaki Y., Hakeem I., Dulaimi A.F., Özkılıç Y., Sabri M., Sergeev V. (2023). Investigation of the physical-mechanical properties and durability of high-strength concrete with recycled PET as a partial replacement for fine aggregates. Front. Mater..

[12] Siddique R., Khatib J., Kaur I. (2008). Use of recycled plastic in concrete: A review. Waste Manag..

[13] Pacheco-Torgal F., Ding Y., Jalali S. (2012). Properties and durability of concrete containing polymeric wastes (tyre rubber and polyethylene terephthalate bottles): An overview. Constr. Build. Mater..

[14] Nikbin I.M., Ahmadi H. (2020). Fracture behaviour of concrete containing waste tire and waste polyethylene terephthalate: An sustainable fracture design. Constr. Build. Mater..

[15] Adamczyk-Królak I. (2018). Guma i politereftalan etylenu z recyklingu—Składniki materiałów (Rubber and polyethylene terephthalate from recycling: Components of building materials). Zesz. Nauk. Politech. Częstochowskiej.

[16] Rakoczy B., Szalewska M., Karpus K. (2014). Prawne Aspekty Zagospodarowania Zasobami Środowiska. Oddziaływanie na Zasoby Środowiska. Towarzystwo Naukowe Organizacji i Kierownictwa.

[17] Raport Roczny PEP 2019. Plastics Europe. https://plasticseurope.org/pl/knowledge-hub/raport-roczny-pep-2019/.

[18] Major M., Major I. (2014). Wykorzystanie odpadów gumowych w budownictwie zrównoważonym (The use of rubber waste in sustainable civil engineering). Bud. O Zoptymalizowanym Potencjale Energetycznym.

[19] Karalar M., Özkılıç Y.O., Aksoylu C., Sabri Sabri M.M., Beskopylny A.N., Stel’makh S.A., Shcherban’ E.M. (2022). Flexural behavior of reinforced concrete beams using waste marble powder towards application of sustainable concrete. Front. Mater..

[20] Özkılıç Y.O., Basaran B., Aksoylu C., Karalar M., Martins C.H. (2023). Mechanical behavior in terms of shear and bending performance of reinforced concrete beam using waste fire clay as replacement of aggregate. Case Stud. Constr. Mater..

[21] Aksoylu C., Özkılıç Y.O., Hadzima-Nyarko M., Isık E., Arslan M.H. (2022). Investigation on improvement in shear performance of reinforced-concrete beams produced with recycled steel wires from waste tires. Sustainability.

[22] Özkılıç Y.O., Karalar M., Aksoylu C., Beskopylny A.N., Stel’makh S.A., Shcherban E.M., Qaidi S., da SA Pereira I., Monteiro S.N., Azevedo A.R.G. (2023). Shear performance of reinforced expansive concrete beams utilizing aluminium waste. J. Mater. Res. Technol..

[23] Batayneh M., Marie I., Asi I. (2008). Promoting the use of crumb rubber concrete in developing countries. J. Waste. Manag..

[24] Girskas G., Nagrockiene D. (2016). The use of steel cord scrap in concrete. Constr. Sci..

[25] Bostanci S.C., Limbachiya M., Kew H. (2016). Portland-composite and composite cement concretes made with coarse recycled and recycled glass sand aggregates: Engineering and durability properties. Constr. Build. Mater..

[26] Thomas B.S., Gupta R.C., Panicker V.J. (2016). Recycling of waste tire rubber as aggregate in concrete: Durability-related performance. J. Clean. Prod..

[27] Siddique R., Naik T. (2004). Properties of concrete containing scrap-tire rubber—An overview. Waste Manag..

[28] Jura J., Pawłowski K. (2017). Ekologiczne aspekty wykorzystania materiałów odpadowych w sektorze budowlanym. Budownictwo Zrównoważone: Wybrane Aspekty Projektowe i Wykonawcze.

[29] Sofi A. (2018). Effect of waste tyre rubber on mechanical and durability properties of concrete—A review. Ain Shams Eng. J..

[30] Batayneh M., Marie I., Asi I. (2007). Use of selected waste materials in concrete mixes. Waste Manag..

[31] Halbiniak J., Katzer J., Major M., Langier B., Major I. (2021). An example of harnessing crushed ceramic pots for the production of watertight concrete. Struct. Concrete.

[32] Rutkowska G., Ogrodnik P., Żółtowski M., Powęzka A., Kucharski M., Krejsa M. (2022). Fly Ash from the thermal transformation of sewage sludge as an additive to concrete resistant to environmental influences in communication tunnels. Appl. Sci..

[33] Katzer J., Halbiniak J., Langier B., Major M., Major I. (2021). Influence of varied waste ceramic fillers on the resistance of concrete to freeze–thaw cycles. Materials.

[34] Halbiniak J., Katzer J., Major M., Major I. (2020). A proposition of an in situ production of a blended cement. Materials.

[35] Yesilata B., Isiker Y., Turgut P. (2009). Thermal insulation enhancement in concretes by adding waste PET and rubber pieces. Constr. Build. Mater..

[36] Mazur W. (2019). Elementy Konstrukcyjne z Ceramiki Budowlanej (Structural Components of Construction Ceramics). Izolacje.

[37] Jaroszewicz M., Klimm J. (2015). Zaawansowane technologie i nowoczesne wyroby ceramiki budowlanej (Advanced technologies and modern building ceramic products). Mater. Ceram..

[38] Grabowski W. (2010). Budownictwo Ogólne: Praca Zbiorowa. T. 1, Materiały i Wyroby Budowlane.

[39] Rzeszutko M. (2008). Ściany akustyczne z pustaków ceramicznych Porotherm. Mater. Bud..

[40] Turgut P., Yesilata B. (2008). Physico-mechanical and thermal performance of newly developed rubber-added bricks. Energy Build..

[41] Fraile-Garcia E., Ferreiro-Cabello J., Defez B., Peris-Fajanes G. (2016). Acoustic behavior of hollow blocks and bricks made of concrete doped with waste-tire rubber. Materials.

[42] Del Coz Diaz J.J., Nieto Garcia P.J., Alvarez Rabanal F.P., Martinez-Luengas A.L. (2011). Design and shape optimization of a new type of hollow concrete masonry block using the finite element method. Eng. Struct..

[43] Dang B.-L., Nguyen-Ngoc H., Duc Hoang T., Nguyen-Xuan H., Abdel Wahab M. (2019). Numerical investigation of novel prefabricated hollow concrete blocks for stepped-type seawall structures. Eng. Struct..

[44] Uenishi K., Shigeno N., Sakaguchi S., Yamachi H., Nakamori J. (2016). Controlled disintegration of reinforced concrete blocks based on wave and fracture dynamics. Struct. Integr. Procedia.

[45] Caruana C., Yousif C., Bacher P., Buhagiar S., Grima C. (2017). Determination of thermal characteristics of standard and improved hollow concrete blocks using different measurement techniques. J. Build. Eng..

[46] Izolacyjność Akustyczna Stropów w Budynkach Mieszkalnych. https://inzynierbudownictwa.pl/izolacyjnosc-akustyczna-stropow-w-budynkach-mieszkalnych/.

[47] Dulak L. (2017). Izolacyjność od Dźwięków Powietrznych i Dźwięków Uderzeniowych Stropów Produkcji.

[48] Müller-Boruttau F. (2009). Ochrona przed drganiami. Elastyczne posadowienie budynków z zastosowaniem materiałów elastomerowych Calenberg—(cz. 1). Przegląd Bud..

[49] Flodr J., Lehner P., Krejsa M. (2020). Experimental and numerical evaluation of clinch connections of thin-walled building structures. Sustainability.

[50] Asphaug S.K., Kvande T., Time B., Peuhkuri R.H., Kalamees T., Johansson P., Berardi U., Lohne J. (2020). Moisture control strategies of habitable basements in cold climates. Build. Environ..

[51] Rouba B.J. (2017). Zawilgocenie jako problem w ochronie obiektów budowlanych i zbiorów muzealnych. Problemy Muzeów Związane z Zachowaniem i Konserwacją Zbiorów.

[52] Trochonowicz M. (2010). Wilgoć w obiektach budowlanych. Problematyka badań wilgotnościowych (Moisture in buildings objects. Humidity testing problems). Bud. I Archit..

[53] Kormanikova E., Kotrasova K., Melcer J., Valaskova V. (2022). Numerical investigation of the dynamic responses of fibre-reinforced polymer composite bridge beam subjected to moving vehicle. Polymers.

[54] Aly A.M., El-Feky M.S., Kohail M., Nasr E.S.A.R. (2019). Performance of geopolymer concrete containing recycled rubber. Constr. Build. Mater..

[55] Kaewunruen S., Li D., Xiang Z. (2018). Enhancement of dynamic damping in eco-friendly railway concrete sleepers using waste-tyre crumb rubber. Materials.

[56] Abdelmonem A., El-Feky M.S., Nasr E.S.A.R., Kohail M. (2019). Performance of high strength concrete containing recycled rubber. Constr. Build. Mater..

[57] Youssf O., El Gawady M.A., Mills J.E. (2015). Experimental investigation of crumb rubber concrete columns under seismic loading. Structures.

[58] Dijckmans A., EKblad A., Smekal A., Degrande G., Lombaert G. (2016). Efficacy of a sheet pile wall as a wave barrier for railway. Soil Dyn. Earthq. Eng..

[59] Kawulok M., Freiherrová N., Horňáková M., Juračka D., Krejsa M. (2023). Hyperbolic paraboloid tensile structure—Numerical CFD simulation of wind flow in RWIND software. Buildings.

[60] Moghadam M.J., Zad A., Mehrannina N., Dasaran N. (2018). Experimental evaluation of mechanically stabilized earth walls with recycled crumb rubbers. J. Rock Mech. Geotech. Eng..

[61] Ismail M.K., Hassan A.A. (2016). Impact resistance and acoustic absorption capacity of self-consolidating rubberized concrete. Mater. J..

[62] Transport—Wyniki Działalności w 2020 Roku. https://stat.gov.pl/obszary-tematyczne/transport-i-lacznosc/transport/transport-wyniki-dzialalnosci-w-2020-roku,9,20.html.

[63] Çelik A.İ., Özkılıç Y.O., Zeybek Ö., Özdöner N., Tayeh B.A. (2022). Performance assessment of fiber-reinforced concrete produced with waste lathe fibers. Sustainability.

[64] Basaran B., Kalkan I., Aksoylu C., Özkılıç Y.O., Sabri M.M.S. (2022). Effects of waste powder, fine and coarse marble aggregates on concrete compressive strength. Sustainability.

[65] Shcherban’ E.M., Stel’makh S.A., Beskopylny A.N., Mailyan L.R., Meskhi B., Shilov A.A., Chernil’nik A., Özkılıç Y.O., Aksoylu C. (2022). Normal-weight concrete with improved stress–strain characteristics reinforced with dispersed coconut fibers. Appl. Sci..

[66] Beskopylny A.N., Shcherban E.M., Stel’makh S.A., Meskhi B., Shilov A.A., Varavka V., Evtushenko A., Özkılıç Y.O., Aksoylu C., Karalar M. (2022). Composition components influence on the concrete properties with the additive of rubber tree seed shells. Appl. Sci..

[67] Major M., Adamczyk-Królak I. (2020). Ażurowy Pustak Ścienny (Openwork Wall Hollow Brick), Politechnika Częstochowska. Patent No.

[68] (2015). Specification for Masonry Units—Part 3: Aggregate Concrete Masonry Units (Dense and Lightweight Aggregates).

[69] (2011). Methods of Test for Masonry Units—Part 16: Determination of Dimensions.

[70] (1996). Eurocode 6—Design of Masonry Structures—Part 1-1: General Rules for Reinforced and Unreinforced Masonry Structures.

[71] (2020). Testing Hardened Concrete—Part 4: Compressive Strength—Specification for Testing Machines.

[72] Technical Data Sheet of SBR Rubber Granules, Orzeł S.A, Poniatowa. www.orzelsa.com.

[73] Technical Data Sheet of Polyethylene Terephthalate in the Form of Colored PET Flakes, PRT Radomsko Sp. z o.o. www.petrecyclingteam.com.

[74] (2017). Technical Specifications, Pustak Betonowy Fundamentowy CJ Blok^®^ PBF-24/12,5.

[75] Gutowski R., Świetlicki W.E. (1986). Dynamika i Drgania Układów Mechanicznych.

[76] Cempel C. (1982). Podstawy Wibroakustycznej Diagnostyki Maszyn.

[77] Cempel C. (1989). Wibroakustyka Stosowana.

[78] Parszewski Z. (1982). Drgania i Dynamika Maszyn.

[79] Mc Duff J.N., Curreri J.R. (1960). Drgania w Technice.

[80] Osiński Z. (1997). Tłumienie Drgań.

[81] Kucharski T. (2018). System Pomiaru Drgań Mechanicznych.

[82] Balaha M.M., Badawy A.A.M., Hashish M. (2007). Effect of using ground waste tire rubber as fine aggregate on the behaviour of concrete mixes. Indian J. Eng. Mater. S..

